# Quantitative evaluation of incomplete preweaning lethality in mice by using the CRISPR/Cas9 system

**DOI:** 10.1038/s41598-018-34270-5

**Published:** 2018-10-30

**Authors:** Takumi Nakamura, Kazuo Nakajima, Tetsuo Ohnishi, Takeo Yoshikawa, Moe Nakanishi, Toru Takumi, Takashi Tsuboi, Tadafumi Kato

**Affiliations:** 1 Department of Life Sciences, Graduate School of Arts and Sciences, The University of Tokyo, 3-8-1 Komaba, Meguro, Tokyo, 153-8902 Japan; 2 Laboratory for Molecular Dynamics of Mental Disorders, RIKEN Center for Brain Science, Wako, Saitama, 351-0198 Japan; 3 Laboratory for Molecular Psychiatry, RIKEN Center for Brain Science, Wako, Saitama, 351-0198 Japan; 4 Laboratory for Mental Biology, RIKEN Center for Brain Science, Wako, Saitama, 351-0198 Japan

## Abstract

Various molecular biology techniques implementing genome editing have made it possible to generate mouse mutants for nearly all known genes; as a result, the International Mouse Phenotyping Consortium (IMPC) database listing the phenotypes of genetically modified mice has been established. Among mouse phenotypes, lethality is crucial to evaluate the importance of genes in mouse survival. Although many genes are reported to show “preweaning lethality, incomplete penetrance” in the IMPC database, the survival rates of homozygous knockout mice are highly variable. Here, we propose the lethal allele index (LAI), the ratio of the observed number of mice with homozygous knockout (KO) to the theoretically predicted number of homozygous KO mice, as a simple quantitative indicator of preweaning lethality. Among the mice mutants registered as incompletely lethal in IMPC, the LAI calculated from the genotypes of F_1_ mice tended to be lower in disease-related genes, and correlated with the frequency of loss-of-function (LOF) alleles in humans. In genome-edited mice using CRISPR/Cas9, the number of mice with homozygous frameshift alleles seemed to be associated with lethality. We edited the *Ehd1* gene in cell lines as well as mice using CRISPR/Cas9, and found that the genotype distribution was significantly different. The LAI calculated from these data was similar to the value calculated from the IMPC data. These findings support the potential usefulness of the LAI as an index of preweaning lethality in genome-edited mice.

## Introduction

The development of genome editing technologies has allowed for the determination of the phenotypes of genetically modified mice for all genes, and databases that accumulate mouse phenotype information, such as the International Mouse Phenotyping Consortium (IMPC, http://www.mousephenotype.org/), have been established^1^. In the IMPC database, 4277 genes have been registered, although many genes remain to be added. Among various phenotypes^1^, “lethality” indicates the importance of a gene during development, with several patterns of lethality registered in the IMPC. Among the 4277 genes in the IMPC database, 411 genes (9.61%) are designated as “preweaning lethality, incomplete penetrance”. The survival rate of homozygous knockout mice designated as “preweaning lethality, incomplete penetrance” is highly variable (Supplementary Table 1).

The CRISPR/Cas9 system is the most useful genome editing tool in mice^2–4^. Compared to the CRISPR/Cas9 system, conventional molecular biology technologies, such as gene targeting methods, require more time to produce genome-edited mice. This is due to their lower recombination efficiency, and the necessity to generate genome-edited embryonic stem (ES) cells before establishing mutant mouse lines^5–7^. In contrast, the CRISPR/Cas9 system can directly cleave the genome in fertilized eggs, and mutant mice can be obtained more effectively^8,9^.

In this study, we propose Lethal Allele Index (LAI), a simple quantitative index, for preweaning lethality. As a proof of concept, we generated the genome-edited mice and cell lines of *EH domain containing 1* (*Ehd1*) gene, which is classified as “preweaning lethality, incomplete penetrance”^10–12^, using CRISPR/Cas9, and assessed the LAI.

## Results

### Possible relationship between lethality and disease-related genes

We defined the Lethal Allele Index (LAI) in a broad sense as the ratio of the observed number of homozygous knockout (KO) mice to the theoretical number of homozygous KO mice predicted using data for other genotypes. When heterozygous KO mice are crossed with each other, the ratio of wild type, heterozygous knockout mice, and homozygous knockout mice of non-lethal genes must be 1:2:1. Thus, we calculated the LAI of genes registered as “preweaning lethality, incomplete penetrance” in IMPC as follows:

$$\begin{array}{rcl}{\rm{Lethal}}\,{\rm{Allele}}\,{\rm{Index}} & = & \frac{{\rm{actual}}\,{\rm{number}}\,{\rm{of}}\,{\rm{homozygous}}\,{\rm{knockout}}\,{\rm{mice}}}{{\rm{theoretical}}\,{\rm{number}}\,{\rm{of}}\,{\rm{homozygous}}\,{\rm{knockout}}\,\mathrm{mice}\,}\\  & = & \frac{{\rm{actual}}\,{\rm{number}}\,{\rm{of}}\,{\rm{homozygous}}\,{\rm{knockout}}\,{\rm{mice}}\times 3}{(\mathrm{actual}\,{\rm{number}}\,{\rm{of}}\,{\rm{wild}}\,{\rm{type}}\,{\rm{and}}\,{\rm{heterozygous}}\,{\rm{knockout}}\,\mathrm{mice})\,}\end{array}$$

**Figure 1:**
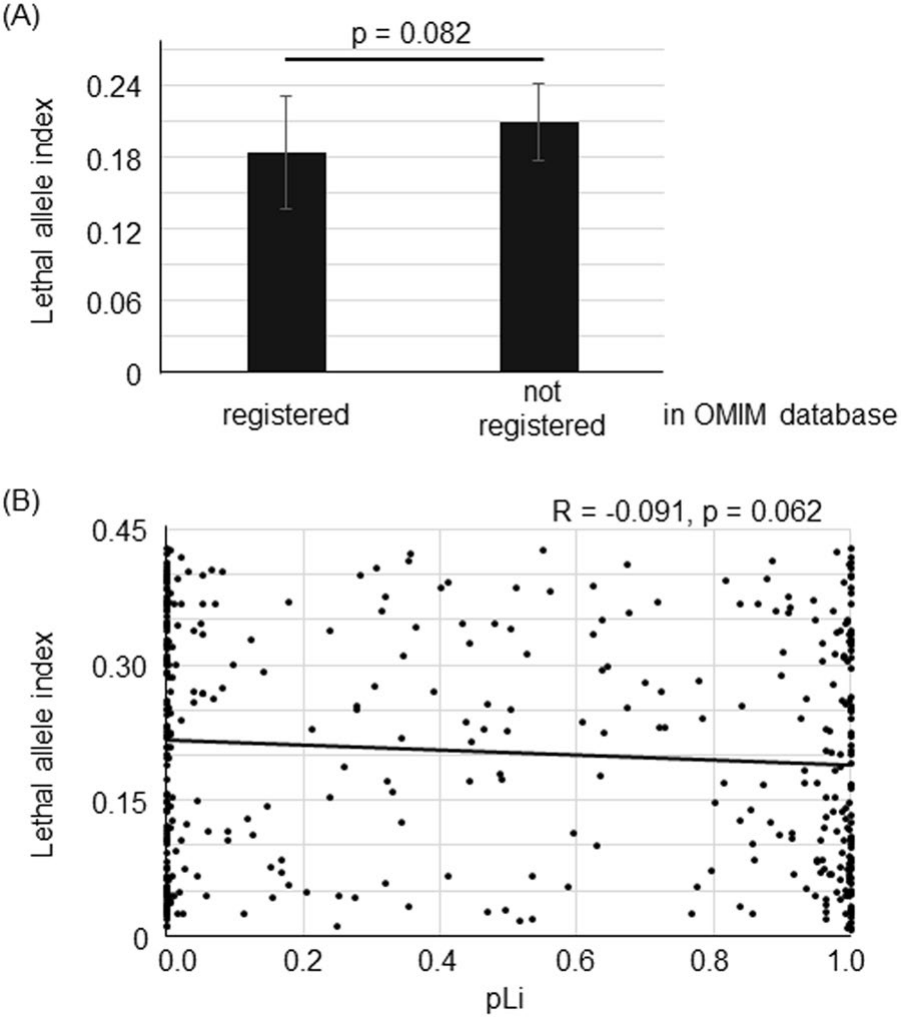
(**A**) The lethal allele index (LAI) of the genes registered as disease-related genes in OMIM tended to be lower than that of genes that were not disease-related (Mann-Whitney U test, p = 0.082, Mean ± SEM, registered; N = 136, not registered; N = 309). (**B**) Lethal allele indexes (LAI) vs pLi. The LAI tended to be correlated with pLi (Pearson’s correlation coefficient, R = −0.091, p = 0.062, N = 423).

We compared the LAI of the genes registered as disease-related genes in the Online Mendelian Inheritance in Man (OMIM) database with the LAI of other genes. The LAI for disease-related genes tended to be lower than that for non-disease related genes (Fig. 1A and Supplementary Table 1, Mann-Whitney U test, p = 0.082).

Next, we compared the LAI with pLi (probability of loss of function intolerance), which is calculated from the genotypes of humans registered in the ExAC (Exome Aggregation Consortium), and indicates the frequency of loss of function mutations in a human population. The LAI showed a tendency of negative correlation with pLi (Fig. 1B and Supplementary Table 1, Pearson’s correlation coefficient, R = −0.091, p = 0.062, N = 423). These findings show that there is a tendency of the LAI to be correlated with clinical significance and loss of function intolerance in humans.

### Genotypes of genome-edited mice generated by CRISPR/Cas9

**Figure 2:**
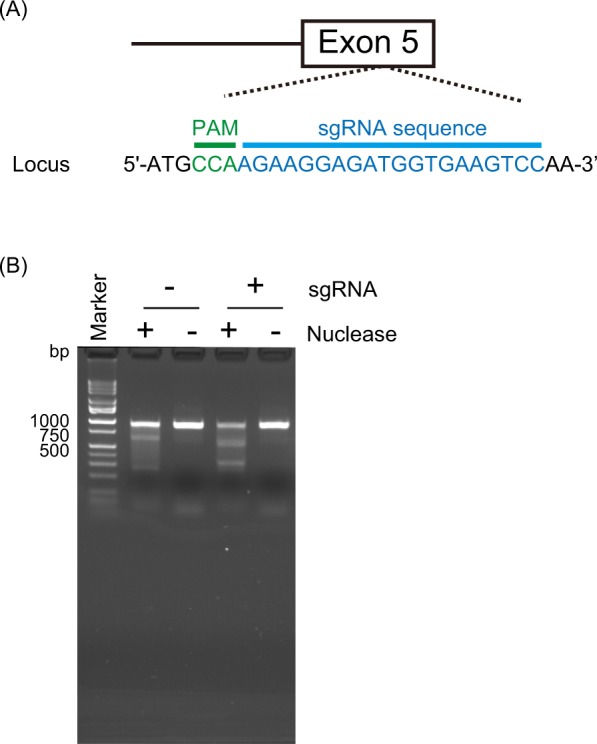
(**A**) Target of CRISPR/Cas9 system in *Ehd1*. The mutation is located in exon 5 of *Ehd1*. (**B**) Surveyor assay. The bands cleaved by nuclease, which recognize mismatches derived from indels by cleavage of Cas9, existed in sgRNA(+)/Nuclease(+).

We generated F_0_ mutant mice of *Ehd1* (Fig. 2) and determined the genotypes of 55 weaned mice from among 79 pups born. The genotype distribution was compared with other lines of genome-edited mice generated by CRISPR/Cas9: *Ntrk1*^13^, *Chdh*^14^, and *Nlgn1*^15^. The rate of the mice homozygous for frameshift alleles to the sum of frameshift /in-frame and in-frame/in-frame genotypes was highest (2.22) for *Chdh*, designated as “viable” in IMPC, lowest (0) for *Ntrk1*, designated as “lethal” in IMPC, and intermediate (0.26) for *Ehd1*, designated as “preweaning lethality, incomplete penetrance” in IMPC, suggesting that the rate of obtaining mice homozygous for loss of function (LOF) alleles reflects the degree of lethality (Table 1).

**Table 1:** Genotypes of *Ehd1* mutant mice and mutant PC12 cells as well as other mutant mice generated by the CRISPR/Cas9 system.

Genotypes^a^	Ehd1		Ntrk1	Chdh	Nlgn1
	PC12 cells^b^	Mice^b^	Mice	Mice	Mice
WT/WT	8	2	0	3	30
WT/in-frame	1	16	0	0	3
WT/frameshift	6	8	24	1	17
in-frame/in-frame	2	14	2	5	0
in-frame/frameshift	12	9	13	4	0
frameshift/frameshift	26	6	0	20	0
Total	55	55	39	33	50
(frameshift/frameshift)/(in-frame/in-frame + in-frame/frameshift)	1.85	0.26	0	2.22	ND
IMPC	—	Incomplete	Lethal	Viable	ND

^a^Mice with other genotypes (knock-in, mosaic, etc.) are not shown in this table. ^b^The genotype distributions of the mice and PC12 cells are significantly different from each other (2 × 6 table, p < 5 × 10^−8^ by Fisher’s exact probability test). ND: not determined.

### Establishment of mutant PC12 cell lines by the CRISPR/Cas9 system

**Figure 3:**
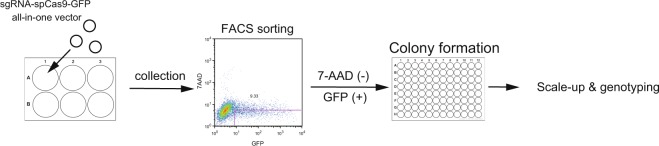
Scheme for establishing *Ehd1* mutant PC12 cells.

To calculate the LAI in the genome-edited mice of *Ehd1* generated by CRISPR/Cas9, we additionally established *Ehd1* mutant cell lines using the CRISPR/Cas9 system. Using these, we determined the ratio of frameshift alleles that can be regarded as LOF alleles to in-frame alleles, regarded as non-LOF alleles. Following transfection of an sgRNA-SpCas9-GFP all-in-one vector, we cloned single green fluorescent protein (GFP)-positive PC12 cells by fluorescence activated cell sorting (FACS). After colony formation, we established and genotyped *Ehd1* mutant PC12 cells (Fig. 3). We performed this cloning step twice, changing the concentration of transfection mixtures in each step. We found that the genotype distribution of frameshift or in-frame indels was significantly different from that in *Ehd1* mutant mice (2 × 6 table, p < 5 × 10^−8^ by Fisher’s exact probability test) (Table 1). The rate of homozygotes of frameshift alleles was significantly smaller in mice (6/55, 10.9%) than cells (26/55, 47.3%) (2 × 2 table, p < 5 × 10^−5^ by Fisher’s exact probability test).

**Figure 4:**
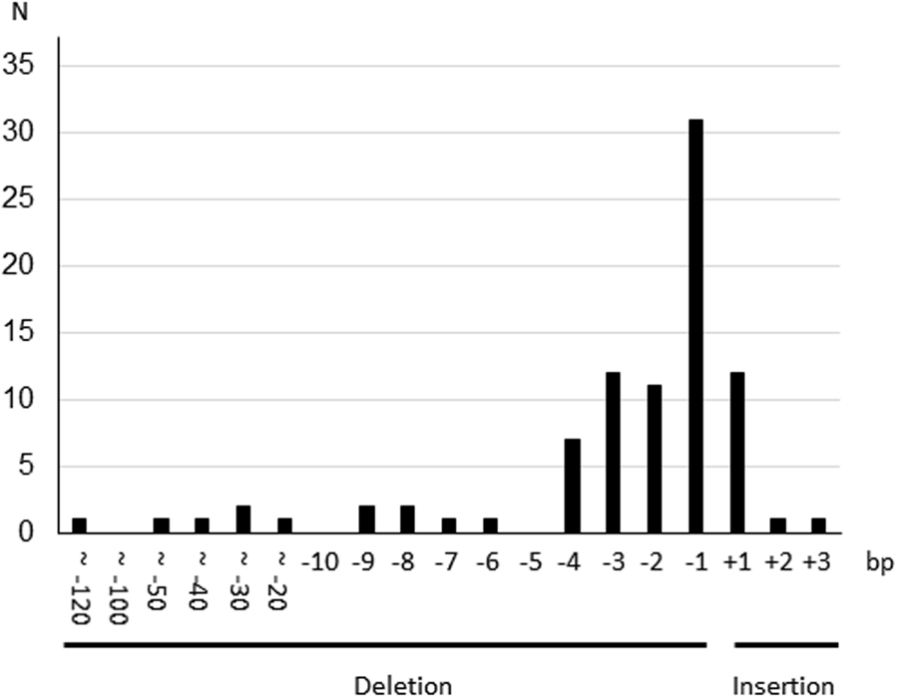
Histogram of cleavage patterns of *Ehd1* mutant PC12 cells. N: number of alleles. bp: base pair.

The distribution of the length of insertion/deletions (indels) is shown in Fig. 4. The rate of frameshift alleles/total cleaved alleles was calculated to be 0.80 (70/87) based on the genotype data of PC12 cells. The expected frequencies of each genotype are shown in Table 2. According to Table 2, the number of homozygous frameshift genotypes (N_f/f_ = 26) and that of homozygous in-frame or heterozygous in-frame/frameshift genotypes (N_i/i_ + N_i/f_ = 2 + 12 = 14) did not significantly deviate from the theoretical ratio of 0.64:0.36 (χ^2^ test for goodness-of-fit, p = 0.89). This suggests that the disruption of *Ehd1* does not confer a disadvantage in the survival of PC12 cells.

**Table 2:** Expected ratio of homozygous cleaved alleles calculated by a cleavage activity based on genotypes of PC12 cells.

	In-frame	Frameshift
In-frame	(1–0.8)^2^ = 0.04	0.8 × (1–0.8) = 0.16
Frameshift	0.8 × (1–0.8) = 0.16	0.80^2^ = 0.64

### Calculation of LAI of *Ehd1* mutant mice

When we define the rate of the frameshift alleles generated by CRISPR/Cas9 as x and denote the frameshift alleles as LOF and in-frame alleles as non-LOF alleles, we can calculate the LAI as follows:

$$\begin{array}{ccc}{\rm{L}}{\rm{A}}{\rm{I}}\,({\rm{C}}{\rm{R}}{\rm{I}}{\rm{S}}{\rm{P}}{\rm{R}}/{\rm{C}}{\rm{a}}{\rm{s}}9) & = & \frac{{\rm{a}}{\rm{c}}{\rm{t}}{\rm{u}}{\rm{a}}{\rm{l}}\,{{\rm{N}}}_{f/f}}{{\rm{t}}{\rm{h}}{\rm{e}}{\rm{o}}{\rm{r}}{\rm{e}}{\rm{t}}{\rm{i}}{\rm{c}}{\rm{a}}{\rm{l}}\,{{\rm{N}}}_{f/f}}=\frac{{\rm{a}}{\rm{c}}{\rm{t}}{\rm{u}}{\rm{a}}{\rm{l}}\,{{\rm{N}}}_{f/f}}{\,({{\rm{x}}}^{2}/(1-{{\rm{x}}}^{2}))({{\rm{N}}}_{i/i}+{{\rm{N}}}_{{\rm{i}}/{\rm{f}}})}\\ {\rm{L}}{\rm{A}}{\rm{I}}(Ehd1) & = & \frac{6}{(0{.8}^{2}/(1-0{.8}^{2}))(14+9)}=0.146\end{array}$$

The LAI calculated from the *Ehd1* genome-edited mice using CRISPR/Cas9 (0.146) was close to the LAI of *Ehd1* (0.111) calculated by the data of IMPC.

## Discussion

In this study, we proposed the LAI as a quantitative index for “preweaning lethality, incomplete penetrance” as the rate of the number of mice with homozygous LOF alleles to the number predicted from the genotypes of other mice. The significant difference in genotypes between *Ehd1* mutant mice and *Ehd1* mutant PC12 cell lines generated by the CRISPR/Cas9 system suggests that the lower rate of mice homozygous for frameshift alleles is due to the lethality of homozygous LOF alleles.

The present result that the homozygous frameshift genotypes of PC12 cells did not show apparent lethality is consistent with a previous report showing that *Ehd1* homozygous knockout murine embryonic fibroblasts (MEFs) can survive^16^. A recent study reported essential genes in humans by identifying genes whose homozygous knockouts were lethal in cell lines^17^. *EHD1* was not included in the list of essential genes, which is also consistent with the present results.

Although we attempted to analyze the genotype distribution of the genome-edited mice by CRISPR/Cas9 for genes designated as “preweaning lethality, incomplete penetrance”, based on previous studies, none of these papers reported on genotype distribution. We believe it would be useful to report on the genotype distribution of F_0_ mice generated by CRISPR/Cas9 as a phenotype of the mutant mice.

In order to calculate the LAI in genome-edited mice by CRISPR/Cas9, we quantified the cleavage pattern, the rate of frameshift alleles to total cleaved alleles, using genome-edited cell lines. Although we performed single cell cloning to establish these cell lines, which took time out of our study and was very costly, it would be enough to perform a transfection of the TA-cloned PCR product from genome-edited cells into competent cells followed by colony PCR and direct sequencing to calculate the rate of frameshift alleles generated by CRISPR/Cas9.

Recently, however, it was reported that CRISPR/Cas9 often causes large deletions near the cut sites^18^, and we thus may have missed large deletions in otherwise apparently “homozygous” genotypes such as WT/WT, in-frame/in-frame, and frameshift/frameshift. Actually, 7 out of the 14 in-frame/in-frame genotypes, and all frameshift/frameshift genotypes, showed single electropherograms, and a possibility that the other allele has a large deletion cannot be ruled out. This fact would not influence the significance of the rate of homozygous frameshift alleles to the sum of frameshift /in-frame and in-frame/in-frame genotypes. In the calculation of LAI, frameshift (LOF)/frameshift (LOF) might be contaminated by frameshift (LOF)/large deletion. If the large deletion is predicted to disrupt genes, that allele should also be considered as an LOF allele. The other value used is “frameshift (LOF)/in-frame + in-frame/in-frame”. In-frame/in-frame may be contaminated by in-frame/large deletion. If large deletion is a LOF mutation, the sum of LOF/in-frame and in-frame/in-frame is not affected by the presence of the large deletion. However, the presence of large deletions affects the ratio of the LOF alleles generated by CRISPR/Cas9 and hampers accurate calculation of LAI. Thus, for the accurate calculation of LAI in genome edited mice using CRIPSR/Cas9, accurate genotyping by long-read sequencing using PacBio platform among others^18^.

The major limitation of this study was the inaccuracy of the LAI of *Ehd1* mutant mice because we did not genotype large deletions using long read sequencing. Nevertheless, the concept of LAI would help to discuss the quantitative evaluation of lethality of genome-edited mice.

## Methods

### Investigation in OMIM database and acquisition of pLi

To investigate whether the “preweaning lethality, incomplete penetrance” genes were registered in the OMIM database, we simply searched the gene name registered in IMPC. The list of pLi was obtained from a previous report^19^. Whenever the gene name registered in IMPC was not found in the list of pLi, alias names registered in the National Center for Biotechnology Information (NCBI) database were verified.

### Establishment of *Ehd1* mutant mice

All animal experiment protocols were approved by the Wako Animal Experiment Committee, RIKEN, and all experiments were performed in accordance with the approved guidelines and regulations. All other experimental procedures were approved by the RIKEN Wako Safety Center and were performed in accordance with the approved guidelines.

We designed a single guide RNA (sgRNA) and a single-strand oligo deoxynucleotide (ssODN) to simulate a single-nucleotide deletion in exon 5 of *EHD1*, identified as a *de novo* mutation in a patient^20^, in mice (Fig. 2A). The mutation introduces a stop codon in the last exon, and is predicted to produce a truncated protein, escaping nonsense-mediated mRNA decay. We generated an sgRNA-Cas9-GFP all-in-one vector and detected its cleavage activity of *Ehd1* in Neuro2A cells in a Surveyor assay (Fig. 2B). We injected a mixture of sgRNA, Cas9 mRNA, and ssODN into fertilized eggs and obtained *Ehd1* mutant mice (F_0_). We performed the injection twice at different concentrations. No knock-in alleles were detected, likely because of the low concentration of ssODN used. Thus, knock-in alleles were not considered in genotype analysis. We excluded undetermined genotypes showing unrecognizable electropherograms.

### Plasmid construction

We generated the sgRNA-SpCas9-GFP all-in-one vector as previously described by Ran *et al*.^2^. The candidate sequences of an sgRNA for *Ehd1* were predicted using CRISPR direct (https://crispr.dbcls.jp/). For the sgRNA oligo insert, 100 pmol of primer set A (Supplemental Table 2A) was annealed and phosphorylated using T4 polynucleotide kinase (New England Biolabs, Ipswich, MA, USA). The reaction conditions were as follows: 37 °C for 30 min, 95 °C for 5 min, and −5 °C/min to 25 °C. The reaction mix was diluted to 1:200 with sterilized water. The diluted reaction mix was inserted into the pSpCas9-2A-GFP (PX458) (#48138, Addgene, Cambridge, MA, USA) vector in the following mixture: 100 ng of pSpCas9-GFP vector, 2 μL of diluted oligo mixture, 1× Fast digest buffer, 0.5 mM DTT, 0.5 mM ATP, and 0.5 μL of Fast digest BbsI and T7 ligase; the volume was brought to 20 μL with sterilized water. Reaction conditions were 6 cycles at 37 °C for 5 min and 21 °C for 5 min. After ligation, residual linearized DNA was excluded using PlasmidSafe ATP-dependent DNase according to the manufacturer’s instructions. Two microliters of the reaction mixture were transformed into 25 μL of NEB Stable Competent *Escherichia coli* (high-efficiency) (C3040, New England Biolabs). Constructs were purified using the PureLink^TM^ HiPure Plasmid Midiprep Kit (Invitrogen, Carlsbad, CA, USA).

### Cell culture

Neuro2A cells and PC12 cells were grown in Dulbecco’s modified Eagle’s medium (D5796, Sigma, St. Louis, MO, USA; 11885, Gibco, Grand Island, NY, USA, respectively) containing the following reagents: for Neuro2A cells, 10% fetal bovine serum (FBS) and 1× Penicillin Streptomycin (PS) (Wako, Osaka Japan, 168–23191) and for PC12 cells, 10% FBS, 10% horse serum (Gibco) and 1× PS mixture.

### Surveyor assay

Neuro2A cells were plated at 8.0 × 10^5^ cells on 6-well plates 24 h before transfection. Aliquots of sgRNA-Cas9-GFP all-in-one vector (3.0 μg) were transfected with 12 μL of Lipofectamine 2000 (Invitrogen), and transfected cells were incubated for 48 h. For genome preparation, the transfected cells were lysed with lysis buffer (10 mM) Tris-HCl (pH 8.0), 150 mM NaCl, 10 mM EDTA, 0.1% SDS, and 1/100 diluted Proteinase K (Roche, Basel, Switzerland), and cell lysates were incubated overnight. The incubated cell lysates were treated with phenol-chloroform extraction followed by ethanol precipitation. Genomic DNA fragments for the surveyor assay were amplified with primer set B (Supplemental Table 2A) and Ex Taq polymerase (Takara, Shiga, Japan) under the following reaction conditions: 96 °C for 2 min, 30 cycles at 94 °C for 30 s and 68 °C for 1 min, and 72 °C for 5 min. The surveyor assay was performed using the Surveyor Mutation Detection Kit (Integrated DNA Technologies).

### Generation of *Ehd1* mutant mice

sgRNA for fertilized eggs injection was transcribed using the MEGAshortscript Kit (Life Technologies, Carlsbad, CA, USA) and the sgRNA-SpCas9-GFP all-in-one vector as a template. A mixture of sgRNA (50 ng/μL), Cas9 mRNA (100 ng/μL, Sigma), and ssODN (first set; 7.9 ng/μL of ssODN_A and second set 50 ng/μL of ssODN_B. Supplemental Table 2B) was microinjected into the cytoplasm of fertilized eggs obtained from C57BL6/N mice. The injected eggs were transferred into the uterus of pseudopregnant ICR females.

### Genotyping of mutant mice

All mutant mice were genotyped by using genomic DNA extracted from tail clips. Tail biopsies were performed on postnatal day 14. Tail clips were incubated in 100 μL of lysis buffer (25 mM NaOH and 0.2 mM EDTA) at 95 °C for 30 min. An equal volume of 40 mM TRIZMA hydrochloride was added to the lysate after incubation, and the mixture was vortexed briefly. Next, 1 μL of tail samples diluted by 2.5-fold using water was amplified by primer set B (Supplemental Table 2A) with Tks Gflex polymerase (Takara) in a 10-μL reaction mix. PCR conditions were as follows: 94 °C for 1 min, 30 cycles at 98 °C for 10 s and 68 °C for 1 min. The PCR products were sequenced with BigDye Terminator V3.1 and an ABI 3730xl sequencer (Life Technologies) using sequencing primers (Supplemental Table 2A).

### Generation of *Chdh* mutant mice

The method for the generation of *Chdh* mutant mice is described elsewhere (Ohnishi *et al*., in submission). In brief, we utilized Cas9 nickase mRNA (CAS504A-1). We injected the Cas9n mRNA and two sgRNAs into fertilized eggs. The sequences of the targets of sgRNAs were as follows: 5′-AGCATGTGGCAGGTCCTTAG-3′ and 5′-CAGTTCCTCTATCCACCGAC-3′.

## Establishement of *Ehd1* mutant PC12 cells by CRISPR/Cas9 System

### Transfection of sgRNA-Cas9-GFP all-in-one vector

sgRNA and ssODN for PC12 cells were also designed to simulate the mutation found in a patient. Because no knock-in alleles were detected, knock-in alleles were not considered in the analysis.

Forty-eight hours before transfection, PC12 cells were plated at 12 × 10^5^ cells per well in 6-well plates. Next, 4.0 μg of sgRNA-Cas9-GFP all-in-one vector and ssODNs (1st set: 10 ng of ssODN_C, D and 2nd set: 100 ng of ssODN_D; Supplemental Table 2B) for mutation knock-in were transfected with 6.0 μL of Lipofectamine 2000 (Invitrogen). ssODN_C was used for mutation knock-in and ssODN_D was used for knock-in of a silent mutation to inhibit additional cutting by Cas9^21^.

### Single-cell sorting by FACS

The transfected cells were treated with 500 μL of 0.025% Trypsin-EDTA (Gibco) for 1.5 min and collected with 4.5 mL 1× PBS followed by centrifugation at 200 × *g* for 3 min. The pellets were suspended in suspension buffer for FACS (2% FBS, 20 mM glucose, and 1**×** PS). The suspension was kept on ice and 7-AAD (BD Biosciences, Franklin Lakes, NJ, USA) was added to distinguish dead cells (5 μL per 1.0 × 10^6^ cells). GFP (+) and 7-AAD (−) PC12 cells were single-cell-sorted to 96-well plates containing 200 μL of conditioned culture medium in each well with a BD FACSAria (BD Biosciences).

### Preparation of conditioned culture medium

The supernatant of the culture medium used to grow confluent PC12 cells was collected. The supernatant was centrifuged for 5 min at 500 × *g* to exclude contaminants. The supernatant was sterilized through a 0.22-μm filter (Merck Millipore, Billerica, MA, USA). The sterilized supernatant was diluted by 10-fold using the normal culture medium (conditioned culture medium).

### Scale-up from single PC12 cell

After single-cell sorting (day 0), the plates were incubated (37 °C and 5% CO_2_) under stationary conditions until day 7. On day 7, a few cells were detected, and 100 μL of conditioned medium were added to each well which had contained 200 μL of conditioned medium. After that, a part of the medium (150 μL) was replaced with new conditioned medium every 4–5 days until the colonies grew to a sufficient size. On day 22, the colonies were split by pipetting and transferred to new 96-well plates. For the passage from the 96-well plate, 60 μL of 0.25% Trypsin-EDTA (Gibco) was added to PC12 cells for 3 min at 37 °C. After incubation, 120 μL of conditioned culture medium was added. Next, 120 and 60 μL of the cell suspension were transferred to a 24-well plate for scale-up and a 96-well plate for genotyping, respectively. In the second independent trial, all the cell suspension was transferred to a 96-well plate for genotyping. Confluent cells cultured on the 24-well plate were trypsinized with 300 μL of 0.25% Trypsin-EDTA (Gibco) for 3 min at 37 °C. After this treatment, trypsin was inactivated by adding 700 μL of conditioned culture medium, and the cells were collected by centrifugation at 200 × *g* for 3 min and transferred into a 6-well plate. Confluent cells in this plate were frozen with Cell Banker 1 plus (Nippon Zenyaku Kogyo, Koriyama, Japan).

### Genotyping of mutant PC12 cells by crude PCR

Genotyping of PC12 cells was performed using crude cell lysates. PC12 cells cultured on 96-well plates were treated with 50 μL of lysis buffer for crude PCR (10 mM Tris-HCl (pH 8.0), 0.1% Triton-X, 10 mM EDTA, and 1/100 diluted proteinase K) at 37 °C overnight. The crude cell lysates were diluted by 10-fold and 1-μL aliquots of the diluted lysates were added to the PCR mix with Ex Taq polymerase (Takara) using primer set C in a total volume of 10 μL (Supplemental Table 2A). Reaction conditions were as follows: 96 °C for 2 min, 30 cycles at 94 °C for 30 s, 63 °C for 30 s, and 72 °C for 30 s, followed by 72 °C for 5 min. PCR fragments were sequenced using the primer set C_Rv (Supplemental Table 2A) with BigDye Terminator V3.1 (Thermo Fisher Scientific, Waltham, MA, USA) and an ABI 3730xl sequencer (Life Technologies). For genotyping, mutation surveyor software (SoftGenetics, State College, PA, USA) was used. An allele containing a deletion of 117 bp was detected in one mutant PC12 cell line, and was included in the frameshift genotype, because such a large deletion can disrupt genes and manifest as a frameshift mutation.

## Electronic supplementary material

Supplementary Information

Supplementary Table 1

Supplementary Table 2
