# Supplementary material for: Quantitative evaluation of incomplete preweaning lethality in mice by using the CRISPR/Cas9 system
Source: Sci Rep. 2018 Oct 30;8:16025. doi: 10.1038/s41598-018-34270-5 (PMC6207718; doi:10.1038/s41598-018-34270-5)
Supplement: Supplementary file 1 — Supplementary Information [file 41598_2018_34270_MOESM1_ESM.docx]

**Supplementary information**

**Quantitative evaluation of incomplete preweaning lethality in mice by using the CRISPR/Cas9 system**

Takumi Nakamura^1, 2^, Kazuo Nakajima^2^, Tetsuo Ohnishi^3^, Takeo Yoshikawa^3^, Moe Nakanishi^4^, Toru Takumi^4^, Takashi Tsuboi^1^ and Tadafumi Kato^2^

^1^ Department of Life Sciences, Graduate School of Arts and Sciences, The University of Tokyo, 3-8-1 Komaba, Meguro, Tokyo 153-8902, Japan

^2^ Laboratory for Molecular Dynamics of Mental Disorders, RIKEN Center for Brain Science, Wako, Saitama 351-0198, Japan

^3^ Laboratory for Molecular Psychiatry, RIKEN Center for Brain Science, Wako, Saitama 351-0198, Japan

^4^ Laboratory for Mental Biology, RIKEN Center for Brain Science, Wako, Saitama 351-0198, Japan

Supplementary Table 1

Supplementary Table 2
